# Clinical analysis and prognostic significance of hepatitis B virus infections for diffuse large B-cell lymphoma with or without rituximab therapy

**DOI:** 10.3892/etm.2013.1079

**Published:** 2013-04-25

**Authors:** WANZHUO XIE, DE ZHOU, KEYUE HU, XIBIN XIAO, WEIJIA HUANG, JINSONG HE, JIMIN SHI, YI LUO, JIE ZHANG, MAOFANG LIN, ZHEN CAI, HE HUANG, XIUJIN YE

**Affiliations:** 1 Department of Hematology, Bone Marrow Transplant Center, The First Affiliated Hospital of Zhejiang University School of Medicine, Hangzhou, Zhejiang 310003;; 2 Department of Hematology, The Second Affiliated Hospital of Zhejiang University School of Medicine, Hangzhou, Zhejiang 310009, P.R. China

**Keywords:** diffuse large B-cell lymphoma, hepatitis B virus, serum hepatitis B virus-DNA load, rituximab, lamivudine

## Abstract

The aim of this study was to analyze the clinical features of hepatitis B surface antigen (HBsAg)-positive and negative diffuse large B-cell lymphomas (DLBCLs) and to compare the outcomes and serum hepatitis B virus (HBV)-DNA loads of patients treated with cyclophosphamide, doxorubicin, vincristine and prednisone (CHOP) regimens with rituximab (RCHOP) or without. A total of 451 DLBCL patients, of which 90 were HBsAg-positive and 361 were HBsAg-negative, were retrospectively reviewed. We compared onset age, gender, Ann Arbor stage, international prognostic index (IPI), lactate dehydrogenase (LDH) and β2-microglobulin (β2-M) levels, as well as overall survival (OS) rates and HBV-DNA loads under CHOP or RCHOP regimens. The OS rate of the HBsAg-positive DLBCL patients was significantly lower than that of HBsAg-negative DLBCL patients and the HBsAg-positive DLBCL patients had an earlier median onset age. HBsAg-positive DLBCL patients had poorer OS rates compared with HBsAg-negative patients (62.2% HBsAg-positive vs. 76.2% HBsAg-negative, P=0.018). HBsAg-positive DLBCL patients with HBV-DNA loads >10^3^ cps/ml during chemotherapy had significantly lower OS rates than those with lower HBV-DNA loads (48.4% HBV-DNA elevated vs. 71.2% HBV-DNA normal, P=0.037). HBsAg-positive DLBCL patients treated with RCHOP had a significantly higher OS rate (79.6%) compared with the 41 CHOP-treated patients (43.9%; P<0.001). HBsAg-positive DLBCL patients with an earlier median onset age and elevated HBV-DNA during chemotherapy had poorer prognoses. HBsAg and HBV-DNA during chemotherapy may be used as prognostic indicators for patients with DLBCL. Rituximab improves the outcome of HBsAg-positive DLBCL patients when administered in combination with anti-viral lamivudine.

## Introduction

Diffuse large B-cell lymphoma (DLBCL) is defined by the World Health Organization (WHO) classification as a heterogeneous entity, encompassing morphologic and genetic variants with variable clinical presentations and outcomes, while constituting the most common type of non-Hodgkin’s lymphoma (NHL) in adults. More than one-third of the world’s population has been infected with the hepatitis B virus (HBV), with the vast majority living in developing regions (1–3). China is a highly endemic HBV area with ∼170 million carriers (4) and previous studies have revealed a high prevalence of HBV infections in NHLs, particularly in DLBCLs (5,6). Several studies have suggested that HBV may even act as an etiological factor for NHL (5,7). However, few studies have focused on the prognosis of hepatitis B surface antigen (HBsAg)-positive DLBCL patients, and the clinical features, prognostic factors and the efficacy of rituximab treatment remain unclear, with no relevant studies comparing the risks and benefits of rituximab application in HBsAg-positive DLBCL patients. Thus, we performed this retrospective study to focus on HBsAg-positive DLBCL patients.

HBV-carrying DLBCL patients are at a higher risk of reactivating hepatitis B with reactivation rates of 20–50% (8) and related mortality rates of 10–40% (9), when receiving cytotoxic chemotherapies, which may be associated with the immunosuppressive effects of the treatments (10). There is a broad range of clinical manifestations of HBV infections, ranging from asymptomatic self-limiting anicteric hepatitis to potentially fatal, severe, progressive liver failure, while two different clinical scenarios contribute to HBV reactivation. Firstly, viral reactivation occurs in patients who suffer from a chronic HBV infection (HBsAg-positive), in whom the diagnosis of HBV reactivation is based on detectable serum HBV-DNA loads in the presence of biochemical or clinical evidence of hepatitis. In the second scenario, viral reactivation occurs in patients who have resolved a HBV infection as shown by the clearance of circulating HBsAg and appearance of antibodies to hepatitis B core antigens (HBcAg) with or without antibodies to HBsAg. In these patients, a low level of HBV replication has been shown to persist in the liver and in peripheral-blood mononuclear cells for decades (11,12). HBV reactivation with the reappearance of HBsAg (HBsAg seroreversion) has been reported following transplantation and immunosuppressive therapy, as well as allogeneic and autologous hematopoietic stem-cell transplantations (13–15).

The cyclophosphamide, doxorubicin, vincristine, and prednisone (CHOP) regimen was once the classical frontline treatment for DLBCL; however, the development of monoclonal antibodies has led to further improvement of DLBCL treatment outcomes. Currently, patients with DLBCL are medicated with an additional immunochemotherapy, usually the rituximab plus CHOP (RCHOP) regimen. Rituximab is a chimeric mouse human monoclonal antibody against CD20^+^, which is an antigen expressed in the majority of B lymphocytes, including malignant lymphomatous B cells and normal B lymphocytes. The incorporation of rituximab into standard chemotherapies has been shown to improve the clinical outcomes of CD20^+^ DLBCL (16,17). However, rituximab, when used alone or in combination with chemotherapies, has been associated with HBV reactivation in patients with DLBCL (18,19). Lamivudine (a reverse-transcriptase inhibitor of the HBV-DNA polymerase) prophylaxis has demonstrated excellent efficacy in the prevention of HBV reactivation for HBsAg-positive patients with DLBCL undergoing conventional chemotherapy, with an excellent safety and tolerability profile (20).

In this retrospective study, we compared the clinical features of DLBCL patients with or without HBV infections and evaluated the potential prognostic factors in HBsAg-positive DLBCL patients. We also compared the treatment outcomes and HBV reactivation rates of patients who received CHOP or RCHOP regimens.

## Materials and methods

### Patients and measurements

We reviewed 536 consecutive newly diagnosed patients with DLBCL who were hospitalized in the First and Second Affiliated Hospitals of the Medical School of Zhejiang University between January 2006 and April 2011. All diagnoses were confirmed by histopathological staining with hematoxylin and eosin (H&E), as well as determination of the immunophenotypes according to the WHO classification. Complete clinical profiles were obtained from 451 patients who received chemotherapy and completed the follow-up. Clinical staging and diagnostic methods included clinical history and physical examinations, chest, abdominal and pelvic computed tomography (CT) scans, digital full-body color Doppler ultrasound diagnosis, marrow aspirate and biopsy, as well as serum lactate dehydrogenase (LDH) and serum β2-microglobulin (β2-M) level measurements (21). Enzyme-linked immunosorbent assays (ELISA) were used to detect HBsAg, hepatitis B surface antibodies (HBsAb), hepatitis B e antigens (HBeAg), hepatitis B e antibodies (HBeAb) and hepatitis B core antibodies (HBcAb).

Routine liver function tests included determinations of alanine aminotransferase (ALT), aspartate aminotransferase (AST), galactosylhydroxylysyl glucosyltransferase (GGT) and bilirubin (direct and indirect forms) levels. These assays were first performed within one week prior to the start of each chemotherapy cycle.

The HBV-DNA loads of all 96 HBsAg-positive patients were determined and an HBV-DNA load >10^3^ cps/ml, which is the upper normal limit in the First Affiliated Hospital of Medical School of Zhejiang University, was regarded as an elevated value. The research was approved by the ethics committee of The First Affiliated Hospital of the Medical School of Zhejiang University and informed consent was obtained from all participants.

### Treatments

Of the 451 patients, for economical reasons, 156 patients were treated only with a CHOP regimen (median number of courses, 6; range, 4–8) and 295 cases were treated with an RCHOP regimen (median number of courses, 6; range, 4–8). Lamivudine prophylaxis was prescribed for all HBsAg-positive patients prior to chemotherapy. Once patients presented an abnormal liver function, they were treated with hepatinica (diammonium glycyrrhizinate, compound glycyrrhizin, reduced glutathione, or polyene phosphatidylcholine) and all chemotherapies were restarted only in cases where the liver function had recovered.

### Follow-up

Following treatment, routine clinical follow-up was conducted every 3 months for the first 2 years and then every 6 months for the additional 3 years. Ultrasound or CT scans were performed every six months during the first two years and then afterwards annually during the follow-up period. The median follow-up time was 27 months (range, 1–76 months).

### Statistical analyses

Significant differences in the baseline clinical parameters and treatment characteristics between the HBsAg-positive patients and HBsAg-negative patients were evaluated by either Chi-square test or Fisher’s exact test for categorical parameters. Overall survival (OS) rates and survival curves were calculated by the Kaplan-Meier method. The OS rate calculation was performed from the date of diagnosis to the date of mortality or the last follow-up. The multivariate analysis of outcome in terms of OS was performed by Cox regression, which included the variables that were significant in a univariate analysis. Two-tailed P-values <0.05 were considered to indicate a statistically significant difference. All statistical analyses were performed with the SPSS software for Windows v.19.0 (SPSS, Inc., Chicago, IL, USA).

## Results

### Clinical characteristics and OS rates of HBsAg-positive and -negative patients

Of the 451 patients, 90 were HBsAg-positive and 361 were HBsAg-negative. The clinical characteristics of these two groups are summarized in Table I. The HBsAg-positive group was associated with an earlier onset age (45 years for HBsAg-positive vs. 56 years for HBsAg-negative, P<0.001), and lower international prognostic index (IPI, 0–2; 63.3% HBsAg-positive vs. 51.2% HBsAg-negative, P=0.040). There were no significant differences between the two groups in gender, Ann Arbor stage, LDH levels, β2-M levels and incidence of B symptoms. In addition, in the HBsAg-positive patient group, rituximab was less applied than in the HBsAg-negative patient group (54.4% HBsAg-positive vs. 68.1% HBsAg-negative, P= 0.015), which may reflect physicians’ concerns about HBV reactivation.

**Figure 1.**
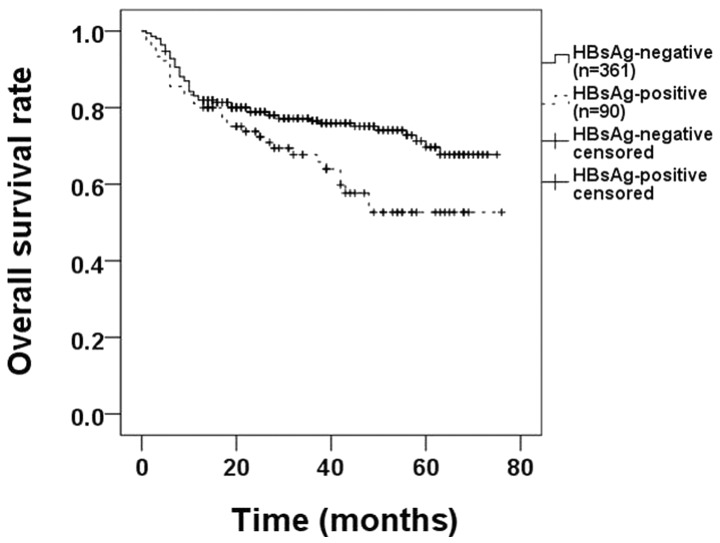
Comparison of OS rates between HBsAg-positive and HBsAg-negative patients with Kaplan-Meier analyses. HBsAg-negative patients had higher OS rates (62.2% HBsAg-positive vs. 76.2% HBsAg-negative; P=0.018). OS, overall survival; HBsAg, hepatitis B surface antigen.

At a median follow-up time of 27 months, we compared the OS rates between HBsAg-positive and HBsAg-negative patients with Kaplan-Meier analyses. HBsAg-negative patients demonstrated a higher OS rate (62.2% HBsAg-positive vs. 76.2% HBsAg-negative, P=0.018; Fig. 1).

### Comparison of OS rates between HBsAg-positive patients with different HBV-DNA loads during chemotherapy

**Figure 2.**
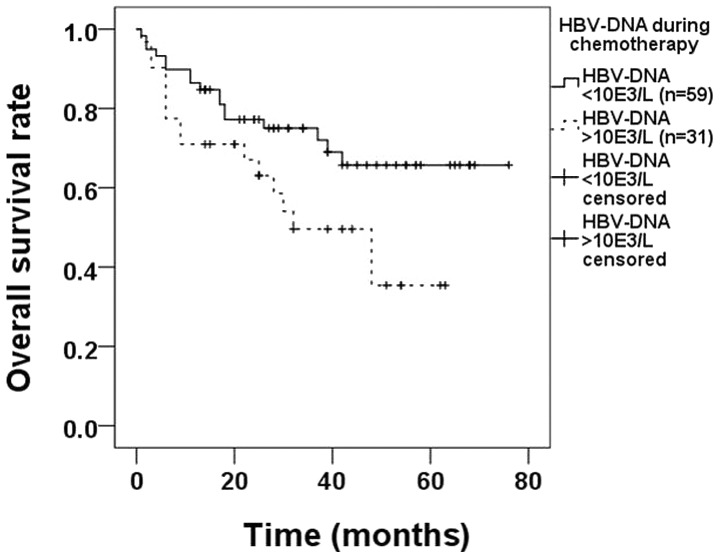
Patients were divided into two subgroups. In the first group the hepatitis B virus (HBV)-DNA was constantly <10^3^ cps/ml during the chemo-therapy, while in the second subgroup, the HBV-DNA was >10^3^ cps/ml for at least one time-point during chemotherapy, resulting in 59 and 31 patients in each group, respectively. The OS in the first group (71.2%) was significantly higher compared with that in the second subgroup (48.4%; P=0.037).

As previously mentioned, the prognosis of HBsAg-positive patients was poorer than that of HBsAg-negative patients. We further investigated whether prognosis was related to the level of HBV activity. We divided patients into two groups according to their HBV-DNA status. In the first group, the HBV-DNA load of the patients was constantly <10^3^ cps/ml during chemo-therapy, while in the second subgroup the HBV-DNA load was >10^3^ cps/ml at least once during chemotherapy; there were 59 and 31 patients in each group, respectively. The OS rate in the first group (71.2%) was significantly higher compared with that in the second subgroup (48.4%, P=0.037; Fig. 2).

### Analysis of prognostic factors for HBsAg-positive DLBCL patients

A univariate analysis revealed that positive B symptoms (P=0.001), Ann Arbor stages III/IV (P=0.002) and elevated LDH levels (P=0.001) were poor prognostic factors for HBsAg-positive DLBCL patients. A multivariate analysis revealed that positive B symptoms [hazard ratio (HR), 14.434; 95% confidence interval (CI), 3.063–68.013; P<0.001), elevated LDH levels (HR, 8.369; 95% CI, 2.059–34.026; P=0.003) and male gender (HR, 0.160; 95% CI, 0.031–0.817; P=0.028) were poor prognostic factors for OS rates (Table II).

### Comparison of therapeutic efficacies and HBV reactivation rates between CHOP and RCHOP treatments

In the 90 HBsAg-positive patients, 41 patients were treated with CHOP (median course, 6; range, 4–8) and 49 patients with RCHOP (median course, 6; range, 4–8). We compared the therapeutic efficacies and the reactivation rates of HBV between the DLBCL patients were treated with CHOP and those who were treated with RCHOP.

**Figure 3.**
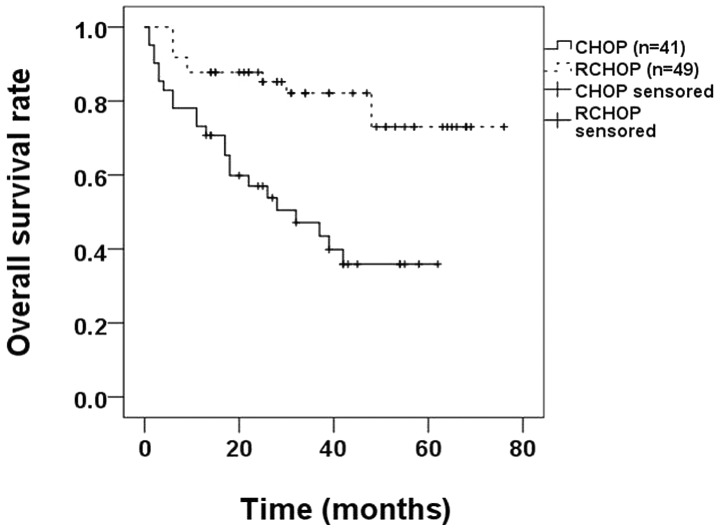
Comparison of the overall survival (OS) rate of the cyclophosphamide, doxorubicin, vincristine and prednisone (CHOP) and rituximab plus CHOP (RCHOP) groups. The OS rate of the RCHOP group (79.6%) was higher compared with that of the CHOP group (43.9%; P<0.001).

For a prognosis evaluation, we used the Kaplan-Meier method to compare the OS rates between the CHOP and RCHOP groups and identified that the OS rate of the RCHOP group (79.6%) was higher compared with that of the CHOP group (43.9%; P<0.0010; Fig. 3).

Regarding HBV-reactivation, we did not identify any patient whose HBsAg status changed from negative to positive following chemotherapy. Therefore, we compared the HBV-DNA loads of the CHOP and RCHOP groups of HBsAg-positive DLBCL patients prior to and during chemotherapy.

Prior to chemotherapy the HBV-DNA level was >10^3^ cps/ml in 22 CHOP (53.7%) and 31 RCHOP (63.3%) patients, without statistical difference between the two groups (P=0.356; Table III).

During chemotherapy, there were 16 patients (39.0%) whose HBV-DNA level was >10^3^ cps/ml in the CHOP group and 15 (30.6%) in the RCHOP group. Due to the anti-viral efficacy of lamivudine, the percentages of patients with HBV-DNA levels >10^3^ cps/ml declined in the two groups when compared with their status before chemotherapy; however, there was no significant difference between the two groups (P=0.403; Table III). Therefore a combined lamivudine application did not bear a greater risk of CHOP-induced HBV reactivation.

## Discussion

HBV infection is a common co-morbidity of DLBCL patients in China. In our study, of the 451 patients, there were 90 HBsAg-positive DLBCL patients and the percentage of HBV carriers was as high as 20.0%, which is higher than the result of a 2006 population census showing that 7.18% of the Chinese population were carrying HBV (22). The present study demonstrated an earlier age of disease onset in the HBsAg-positive DLBCL patient group. These two facts suggest that HBV acts as an etiologic factor for DLBCL development, which is in agreement with the results of an earlier relevant study; however, an apparent correlation between HBsAg and prognosis was not revealed in the earlier study (23). The current study demonstrated that HBsAg-positive DLBCL patients had poorer OS rates compared with HBsAg-negative patients (62.2% HBsAg-positive vs. 76.2% HBsAg-negative, P=0.018; Fig. 1). One possible reason for the different conclusion of former scholars is that in our study the percentage of patients receiving rituximab in the HBsAg-negative group was higher compared with that in HBsAg-positive group. To address whether HBV infection is another cause for a worse prognosis in HBsAg-positive DLBCL patients, we further divided the HBsAg-positive DLBCL patients into two groups according to the HBV-DNA loads during chemotherapy. We identified that patients with HBV-DNA loads >10^3^ cps/ml during chemotherapy had poorer prognoses compared with patients with HBV-DNA loads <10^3^ cps/ml (71.2% HBV-DNA normal vs. 48.4% HBV-DNA elevated, P=0.037; Fig. 2) and we suggest that in HBsAg-positive DLBCL patients, the poor management of HBV was related to poor prognosis. Therefore, we suggest that a more rigorous anti-viral management should be administered to patients whose HBV-DNA titer is >10^3^ cps/ml, despite a standard anti-viral treatment during chemotherapy.

The results of the current study suggest that HBsAg and HBV-DNA loads during chemotherapy may be used as prognostic indicators for patients with DLBCL. A univariate analysis revealed that positive B symptoms (P=0.001), Ann Arbor stages III/IV (P=0.002) and elevated LDH levels (P=0.001) were poor prognostic factors for HBsAg-positive DLBCL patients and a multivariate analysis revealed that positive B symptoms (P<0.001), elevated LDH levels (P=0.003) and male gender (P=0.028) were poor prognostic factors for the OS rates in HBsAg-positive DLBCL patients.

We compared therapeutic efficacies and HBV reactivation rates between CHOP and RCHOP treatments in the present study. Regarding HBV-reactivation, we did not identify any patient whose HBsAg status changed from negative to positive following chemotherapy. We also compared the CHOP and RCHOP groups’ serum HBV-DNA values of HBsAg-positive DLBCL patients before and during chemotherapy and identified that there was no significant difference between the two groups (Table III), indicating that an RCHOP regimen did not bear a greater risk of HBV reactivation compared with a CHOP regimen when combined with a lamivudine anti-viral treatment. In addition, for HBsAg-positive DLBCL patients, the OS rate of patients receiving RCHOP was 79.6%, which is higher than than the 43.9% for patients receiving CHOP (P<0.001; Fig. 3), suggesting that HBsAg-positive DLBCL patients benefit from the additional rituximab application.

Since RCHOP has an advantage over CHOP on the OS rate and since the management of lamivudine with the RCHOP regime did not increase the risk of HBV reactivation, our study demonstrated that a RCHOP treatment was appropriate for HBsAg-positive patients and resulted in a better prognosis.

## Figures and Tables

**Table I.** Clinical characteristics of HBsAg-positive and negative patients.

	HBsAg-positive patients (n=90, 20%)	HBsAg-negative patients (n=361, 80%)	P-value
Median age (years)	45	56	<0.001
Age range	18–69	18–83	
Gender			
Male	58 (64.4%)	197 (54.6%)	0.091
Female	32 (35.6%)	164 (45.4%)	
Stage			
I/II	16 (17.8%)	69 (19.1%)	0.772
III/IV	74 (82.2%)	292 (80.9%)	
IPI			
0–2	57 (63.3%)	185 (51.2%)	0.040
3–5	33 (36.7%)	176 (48.8%)	
LDH			
>ULN (225 U/l)	44 (48.9%)	159 (44.0%)	0.409
Normal	46 (51.1%)	202 (56.0%)	
β2-M			
>ULN (2200 *μ*g/l)	32 (35.6%)	161 (44.6%)	0.121
Normal	58 (64.4%)	200 (55.4%)	
B symptoms			
Yes	28 (31.1%)	121 (33.5%)	0.664
No	62 (68.9%)	240 (66.5%)	
Regimen			
CHOP	41 (45.6%)	115 (31.9%)	0.015
RCHOP	49 (54.4%)	246 (68.1%)	

HBsAg, hepatitis B surface antigen; LDH, lactate dehydrogenase; ULN, upper level of normal; β2-M, β2-microglobulin; IPI, international prognostic index; CHOP, cyclophosphamide, doxorubicin, vincristine and prednisone; RCHOP, rituximab plus CHOP.

**Table II.** Univariate and multivariate analyses of clinical factors for OS rates of HBsAg-positive DLBCL patients.

Variables	OS					
	Univariate analysis			Multivariate analysis		
	HR	95% CI	P-value	HR	95% CI	P-value
Age >60 years	0.889	0.203–3.899	0.876	0.705	0.144–3.441	0.665
Gender, male	0.662	0.308–1.427	0.293	0.160	0.031–0.817	0.028
Ann Arbor stage III/IV	9.071	1.240–66.340	0.002	9.729	0.991–95.528	0.051
Positive B symptoms	3.256	1.657–6.399	0.001	14.434	3.063–68.013	<0.001
LDH level >ULN (225 U/l)	3.863	1.745–8.549	0.001	8.369	2.059–34.026	0.003
β2-M >ULN (2200 *μ*g/l)	1.019	0.504–2.059	0.959	0.353	0.108–1.151	0.084

OS, overall survival; HR, hazard ratio; CI, confidence interval; HBsAg, hepatitis B surface antigen; DLBCL, diffuse large B-cell lymphoma; LDH, lactate dehydrogenase; β2-M, β2-microglobulin; ULN, upper level of normal.

**Table III.** HBV reactivation comparisons of DLBCL patients treated with CHOP or RCHOP.

	CHOP group (n=41, 20.3%)	RCHOP group (n=49, 79.7%)	P-value
HBV-DNA >10^3^ cps/ml before chemotherapy	22 (53.7%)	31 (63.3%)	0.356
HBV-DNA >10^3^ cps/ml during chemotherapy	16 (39.0%)	15 (30.6%)	0.403

HBV, hepatitis B virus; DLBCL, diffuse large B-cell lymphoma; CHOP, cyclophosphamide, doxorubicin, vincristine and prednisone; RCHOP, rituximab plus CHOP.
